# Using GRADE Evidence to Decision frameworks to support the process of health policy-making: an example application regarding taxation of sugar-sweetened beverages

**DOI:** 10.1093/eurpub/ckac077

**Published:** 2022-11-29

**Authors:** Julia Stadelmaier, Eva A Rehfuess, Sarah Forberger, Angelika Eisele-Metzger, Blin Nagavci, Holger J Schünemann, Joerg J Meerpohl, Lukas Schwingshackl

**Affiliations:** Institute for Evidence in Medicine, Medical Centre - University of Freiburg, Faculty of Medicine, University of Freiburg, Freiburg, Germany; Institute for Medical Information Processing, Biometry and Epidemiology (IBE), LMU Munich, Munich, Germany; Pettenkofer School of Public Health, Munich, Germany; Department of Prevention and Evaluation, Leibniz-Institute for Prevention Research and Epidemiology - BIPS, Bremen, Germany; Institute for Evidence in Medicine, Medical Centre - University of Freiburg, Faculty of Medicine, University of Freiburg, Freiburg, Germany; Cochrane Germany, Cochrane Germany Foundation, Freiburg, Germany; Institute for Evidence in Medicine, Medical Centre - University of Freiburg, Faculty of Medicine, University of Freiburg, Freiburg, Germany; Department of Health Research Methods, Evidence and Impact, McMaster University, Hamilton, Canada; Department of Medicine, McMaster University, Hamilton, Canada; Institute for Evidence in Medicine, Medical Centre - University of Freiburg, Faculty of Medicine, University of Freiburg, Freiburg, Germany; Cochrane Germany, Cochrane Germany Foundation, Freiburg, Germany; Institute for Evidence in Medicine, Medical Centre - University of Freiburg, Faculty of Medicine, University of Freiburg, Freiburg, Germany

## Abstract

**Background:**

Grading of Recommendations, Assessment, Development and Evaluation (GRADE) Evidence to Decision (EtD) frameworks are well-known tools that enable guideline panels to structure the process of developing recommendations and making decisions in healthcare and public health. To date, they have not regularly been used for health policy-making. This article aims to illustrate the application of the GRADE EtD frameworks in the process of nutrition-related policy-making for a European country.

**Methods:**

Based on methodological guidance by the GRADE Working Group and the findings of our recently published scoping review, we illustrate the process of moving from evidence to recommendations, by applying the EtD frameworks to a fictitious example. Sugar-sweetened beverage (SSB) taxation based on energy density was chosen as an example application.

**Results:**

A fictitious guideline panel was convened by a national nutrition association to develop a population-level recommendation on SSB taxation aiming to reduce the burden of overweight and obesity. Exemplary evidence was summarized for each EtD criterion and conclusions were drawn based on all judgements made in relation to each criterion. As a result of the high priority to reduce the burden of obesity and because of the moderate desirable effects on health outcomes, but considering scarce or varying research evidence for other EtD criteria, the panel made a conditional recommendation for SSB taxation. Decision-makers may opt for conducting a pilot study prior to implementing the policy on a national level.

**Conclusions:**

GRADE EtD frameworks can be used by guideline panels to make the process of developing recommendations in the field of health policy more systematic, transparent and comprehensible.

## Introduction

Policy-makers in the health sector and beyond often have to set priorities and make health-related decisions on behalf of a population; these policy-makers comprise elected politicians as well as decision-makers in technical agencies. A relevant tool to support policy-makers in healthcare and public health are evidence-based, trustworthy guidelines, which contain recommendations for policy actions.^1^

Health policy actions are defined as actual options taken and pursued by policy-makers; they may be part of broader strategies or action plans (policies) which aim to achieve the best possible health for the general population.^2^^,^^3^

Using evidence-based methods in health policy-making is complex.^4^ Health policies on a population level, such as sugar-sweetened beverage (SSB) taxation, affect large numbers of people and different social groups. Thus, they should not only depend on the magnitude of anticipated health benefits and potential harms for a target population, but also on the certainty of intervention effects, the value people place on the associated outcomes - whether desirable or undesirable - and interventions, and the impact on resources or cost savings. In addition, feasibility, acceptability and equity are important factors to consider.^5^ Moreover, when taking a complex systems perspective, interactions between different parts of an intervention (or strategy), between different parts of the system (e.g. individual and societal levels) and between the intervention and the system (e.g. adaption processes) have to be considered. The introduction of a tax on SSBs, for example, may lead to reformulation of existing products, changes in marketing strategies or price changes in products other than SSBs and thus changes in consumption and in anticipated health effects.^6^ When developing, adopting, implementing or evaluating health policies it is therefore necessary to ensure that all relevant criteria are taken into consideration, and that the best available evidence for the target populations is sought.^5^

The Grading of Recommendations, Assessment, Development and Evaluation (GRADE) approach is the most widely used approach for rating the outcome-specific certainty of evidence and determining the strength of recommendations.^7^ The GRADE Evidence to Decision (EtD) frameworks were developed to enable guideline panels and decision-makers to structure the process of moving from evidence to decisions, while considering relevant *a priori* agreed criteria from different viewpoints. These frameworks comprise sets of criteria as well as procedural guidance and intend to ensure that the best available evidence is considered, and the underlying rationale is made explicit and transparent.^8^ GRADE EtD frameworks were originally developed for clinical recommendations, and gradually adapted to other types of recommendations and decisions, such as coverage decisions, health system recommendations and most recently to health policy-making.^5^^,^^8^

Recently, we conducted a scoping review on how and to what extent the GRADE approach has already contributed to policy development in the context of nutrition and physical activity interventions on health outcomes.^9^ This research was undertaken within the Policy Evaluation Network (PEN), a consortium funded by the European Union that was set up to assess and improve implementation and evaluation of policies in the areas of physical activity, dietary behaviour and sedentary behaviour in Europe.^3^ We found that GRADE EtD frameworks might be suitable for health policy development but have so far not been used much. Overall, we identified only four health policy guidelines published by the World Health Organization (WHO) which used GRADE EtD frameworks (e.g. sodium intake in children and adults; Supplementary table S1). The use of GRADE EtD in those guidelines is suboptimal^9^ and not in line with current GRADE guidance.^5^^,^^8^ Moreover, in our survey conducted among European health policy-makers, respondents agreed to be familiar with GRADE and expressed interest of using GRADE for evidence-based policy-making.^9^ Since developing trustworthy recommendations is a key step in policy-making and GRADE is used by more than 100 organizations worldwide,^8^ elaborating an example application of GRADE EtD for the area of health policy-making seems to be reasonable. This is especially important for policy- and decision-makers, but also for guideline organizations, developers and users, since GRADE EtD support a structured and transparent process of developing recommendations.

Thus, this article illustrates the application of the GRADE EtD frameworks to a selected health policy, using the development of a recommendation on SSB taxation as a fictitious application example.

## Methods

### Development process of recommendations for or against a health policy

The GRADE EtD frameworks are composed of three sections that reflect key steps in the process of moving from evidence to a decision (e.g. recommendation for a health policy): (i) formulating the question, (ii) making an evidence-informed assessment and (iii) drawing conclusions.^5^

**Figure 1:**
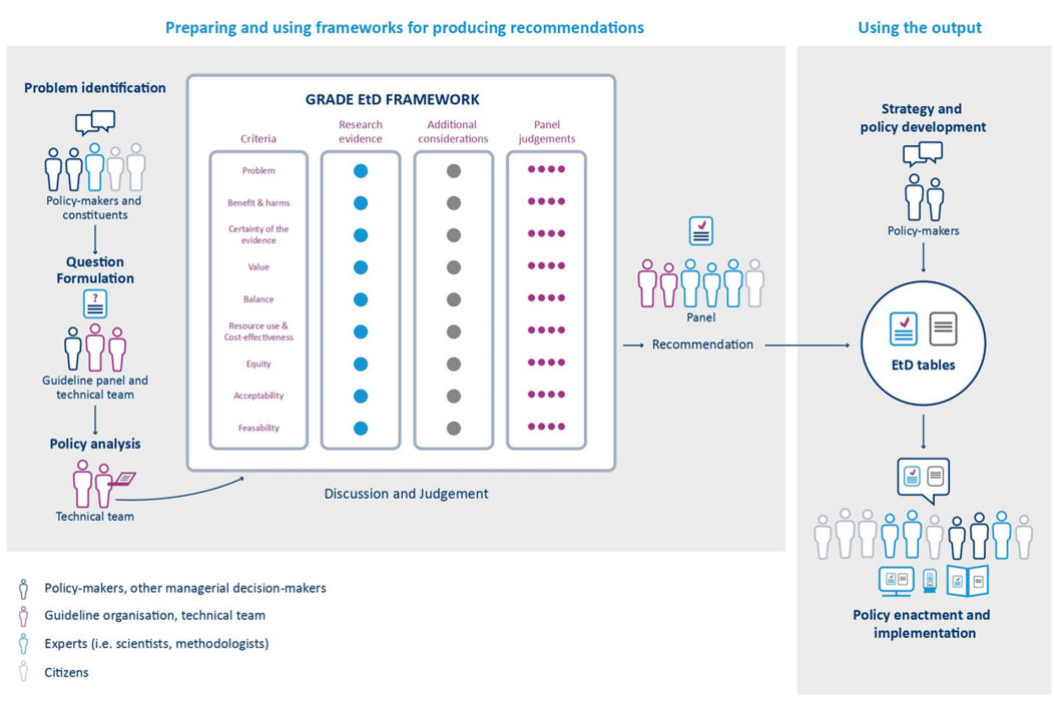
Evidence to Decision conceptual map workflow adapted for policy-making (adapted from Moberg *et al*.)^5^

Figure 1 shows a conceptual workflow for the EtD frameworks in the three-step process of developing recommendations for a health policy. In the first step, a professional health association convenes a guideline panel, usually comprising scientists with expertise in the fields relevant to the policy decision at hand, representatives of those affected by the policy, and methodologists. For example, panel members may be recruited from working groups and scientific advisory boards of the professional health association that coordinates the guideline. The composition of the panel is discussed against the background of relevance and degree of being affected. Financial and intellectual conflicts of interest of each panel member need to be defined and addressed (e.g. when including the sugar industry). The panel members describe the scope of the guideline by defining the health problem, and policy options to address it. In the context of reducing obesity and its associated health and economic consequences, for instance, food policy strategies may be based on fiscal incentives (e.g. SSB taxation), population education (e.g. 5-a-day fruit and vegetables campaign), point-of-purchase labelling (e.g. restaurant calorie labelling) or procurement and nutrition standards.^10^ The guideline panel often works with a technical team to formulate questions. The PICO scheme (Population, Intervention, Comparator, Outcome) is highly recommended for formulating the research questions.

In the second step, the technical team searches for and summarizes the best available evidence (e.g. by systematically reviewing the published scientific literature or generating new evidence syntheses). It uses the GRADE EtD frameworks to consider evidence for each criterion and provide any information for additional consideration by the panel, with links to more detailed information. Panel members are informed with a concise summary of the best available evidence about the relative pros and cons of the policies with regards to nine pre-defined criteria: priority of the problem, benefits and harms, certainty of the evidence, values, balance of effects, resources required and cost-effectiveness, equity, acceptability and feasibility (Supplementary table S2). A detailed description of these EtD criteria can be found elsewhere.^5^^,^^8^^,^^11^

In the third step, the panel discusses these findings and makes judgements in relation to each criterion. Finally, panel members draw conclusions based on all judgements and make a strong or conditional recommendation for or against a health policy. They provide a summary of their recommendations (EtD table), together with other key issues for implementation, e.g. notes about monitoring and evaluation or research gaps. EtD tables are published (e.g. in reports or journal articles) and, ideally, benefit from public consultation, by receiving feedback from different stakeholders (e.g. professional associations, interest groups). Policy-makers can use EtD tables for an evidence-informed discussion and decision on the implementation (fully or partially), adaptation or rejection of a policy.^5^

### Setting and topic for example application: sugar-sweetened beverage taxation

In our application example on using the GRADE EtD frameworks for an SSB tax, we take the perspective of a national nutrition association with a policy mandate in a high-income European country that has not imposed a tax on SSBs yet. Our exemplary country is characterized by very high and steadily increasing rates of overweight and obesity in the child and adult population. The aim of the health policy is the prevention of overweight and obesity with the overarching goal to reduce the burden of non-communicable diseases (NCDs).

Based on the findings of our scoping review and guidance from the GRADE Working Group (Supplementary appendix S1), we illustrate the example with the three-step process described above. First, we provide research evidence about the problem and its priority, and present general research questions, on which the subsequent work of the technical team is based. Second, for each of the aforementioned EtD criteria, we present exemplary evidence, which we identified either through our scoping review, or our search for relevant studies (i.e. high-quality systematic reviews, modelling studies) in PubMed (e.g. keywords: *systematic review* AND *sugar-sweetened beverage taxation*) until 10 December 2021. Moreover, we used data from modelling studies, guidelines by health organizations, and cost-effectiveness analyses or other individual studies if no systematic reviews were available. Finally, we assume judgements of the fictitious panel for each criterion and conclude with a fictitious overall recommendation.

## Results

### Formulating the question

The exemplary nutrition association of the exemplary country convened a fictitious panel of 12 experts comprising 2 methodologists, 5 health scientists (1 epidemiologist, 1 public health specialist, 2 nutritionists and 1 sociologist), 2 physicians, 1 ethicist and 2 economists. For none of the panel members a relevant conflict of interest was identified (Supplementary table S3).

Based on the discussion, the fictitious panel concluded that reducing SSB intake by introducing taxes on SSBs is a priority, since the health consequences of obesity are significant for individuals but also for the society and the health system. Moreover, obesity prevention is crucial for achieving the sustainable development goal target 3.4 to reduce by one-third the premature mortality from NCDs by 2030.^12^ Thus, the following question was formulated: What is the impact of introducing a tax on SSBs compared to no taxation? The detailed description of the PICO scheme is presented in table 1 and Supplementary table S4.

**Table 1:** Description of population (P), intervention (I), comparison (C) and outcomes (O)

Population	General population of a European high-income country. Children/adolescents, patients with overweight or obesity, and disadvantaged groups (e.g. with low socioeconomic status) may be relevant subgroups to consider.
Intervention	Taxation on SSB: The definition of SSB covers drinks with added sugars, such as sodas, fruit drinks, energy or sports drink or sugar-sweetened tea. The amount of taxation is determined by the energy density (grams of sugar per 100 ml of drink). The tax will be applied with a rate of 20% for drinks with more than 5 g of sugar per 100 ml.
Comparison	No taxation.
Outcomes	Anthropometric outcomes, e.g. risk of overweight/obesity, changes in body weight (kg), BMI (kg/m^2^). Non-communicable diseases, e.g. cardiovascular disease, type 2 diabetes. Dietary behaviour outcomes, e.g. SSB intake (ml/day), total energy consumed (kcal/day). Economic parameters, e.g. implementation costs, tax revenues ($).

BMI, body mass index; SSB, sugar-sweetened beverages.

### Making an evidence-informed assessment

In the following sections, exemplary research evidence for each EtD criterion is reported, and a short summary of the judgement for each EtD criterion is given in table 2. A detailed description of the EtD tables can be found in Supplementary table S5.

**Table 2:** Description of the GRADE Evidence to Decision (EtD) criteria for the fictitious sugar-sweetened beverage taxation in the prevention of obesity and its associated non-communicable diseases

Problem	
Is the problem a priority?	
JUDGEMENT	RESEARCH EVIDENCE
No Probably no Probably yes ***Yes*** Varies Don't know	Burden of NCDs is the leading cause of death worldwide. Obesity, hypertension, dyslipidaemia and high blood glucose are major risk factors for the development of NCDs. Half of the adult population in the corresponding European country is overweight, and one-fifth is obese. High intake of SSB is strongly associated with the morbidity of NCDs. Intake of SSB is high in the corresponding European country.
Desirable effects	
How substantial are the desirable anticipated effects?	
JUDGEMENT	RESEARCH EVIDENCE
Trivial Small ***Moderate*** Large Varies Don't know	Systematic reviews showed favourable effects on dietary intake (reduction in SSB intake), and a lower BMI for a 10% price increase in SSB. One systematic review indicated also a lower prevalence of overweight and obesity. SSB tax may be particularly effective, when the tax is high, specific for beverage volume, and it is applied to a broad definition of SSB.
Undesirable effects	
How substantial are the undesirable anticipated effects?	
JUDGEMENT	RESEARCH EVIDENCE
Large Moderate Small Trivial ***Varies*** Don't know	No systematic review is available on the unintended or adverse effects of SSB taxation. Individuals may compensate less intake of SSB by consuming other unhealthy foods. Manufacturer may only reformulate beverages by replacing sugar with non-nutritive sweeteners. Decline in employment in the SSB industry may be offset by the creation of new jobs in the non-SSB industry.
Certainty of evidence	
What is the overall certainty of the evidence of effects?	
JUDGEMENT	RESEARCH EVIDENCE
Very low ***Low*** Moderate High No included studies	The certainty of evidence and net benefit of lower SSB consumption was rated as moderate, whereas the rating for the net benefit reduction in BMI was small. Low certainty of the evidence for a reduced consumption of taxed sugar-added foods. Dose–response meta-analyses: each daily serving increase in SSB was associated with higher risk of type 2 diabetes (high certainty), all-cause mortality (low certainty), cardiovascular disease (moderate certainty), hypertension (low certainty) and obesity (low certainty).
Values	
Is there important uncertainty about or variability in how much people value the main outcomes?	
JUDGEMENT	RESEARCH EVIDENCE
Important uncertainty or variability Possibly important uncertainty or variability Probably no important uncertainty or variability ***No important uncertainty or variability***	Weight loss is reported to be an important outcome, since 50% of population tried to reduce body weight in the previous 12 months. NCDs such as coronary heart disease and stroke are the main causes of disability worldwide, and obesity a key risk factor. Obesity and NCDs can have a major impact in the life not only of the person affected but also of the close network (family and friends), as well as their caregivers. Lower costs for public healthcare services and social welfare systems are of public interest. There is no reason to believe there is important uncertainty about or variability in outcome importance.
Balance of effects	
Does the balance between desirable and undesirable effects favour the intervention or the comparison?	
JUDGEMENT	RESEARCH EVIDENCE
Favours the comparison Probably favours the comparison Does not favour either the intervention or the comparison ***Probably favours the intervention*** Favours the intervention Varies Don't know	Please see research evidence on desirable and undesirable effects above; and certainty of the evidence.
Resources required	
How large are the resource requirements (costs)? What is the certainty of the evidence of resource requirements (costs)?	
JUDGEMENT	RESEARCH EVIDENCE
Large costs Moderate costs Negligible costs and savings ***Moderate savings*** Large savings Varies Don't know Certainty of evidence Very low Low Moderate High ***No included studies***	The EtD criteria ‘resources required’ was interpreted as: additional taxes generated by the country. Modelling study in South Africa: a 10% tax on SSB would result in $450 million dollars of tax revenues. Modelling study in Canada: a 20% tax on SSBs would result in overall revenues of $1.1 billion per year.
Cost effectiveness	
Does the cost-effectiveness of the intervention favour the intervention or the comparison?	
JUDGEMENT	RESEARCH EVIDENCE
Favours the comparison Probably favours the comparison Does not favour either the intervention or the comparison ***Probably favours the intervention*** Favours the intervention Varies No included studies	SSB tax was highly cost-saving due to health gains (24 times the tax-implementation costs). Modelling study in South Africa: a 10% increase in the tax would prevent about 8000 type 2 diabetes-related premature deaths over a period of 20 years. Modelling study in Canada: a 20% tax on SSBs would result in an overall tax burden of $1.1 billion per year ($30 to $35 per person) and direct healthcare savings at $1.7 to $2.0 billion per quintile lifetime, depending on the income group. Microsimulation study: health savings in sugar content taxes are about twice as large as volume based taxes.
Equity	
What would be the impact on health equity?	
JUDGEMENT	RESEARCH EVIDENCE
Reduced Probably reduced Probably no impact ***Probably increased*** Increased Varies Don't know	Individuals with a lower socioeconomic status are at risk of consuming more SSB, and are at higher risk for NCDs. Increasing the price of SSB has an impact on health, particularly among individuals with a low socioeconomic status, and therefore contributes to the reduction of health inequalities. Allocating revenues to projects that address the needs of disadvantaged groups or impacted communities can also promote health equity.
Acceptability	
Is the intervention acceptable to key stakeholders?	
JUDGEMENT	RESEARCH EVIDENCE
No Probably no Probably yes Yes ***Varies*** Don't know	Mixed-method systematic review indicated that 39–66% of the general public support an SSB tax, but political acceptability was not estimable. SSB taxes tend to be supported more by people with a high socioeconomic level, low SSB consumption, normal weight status and no children at home. Survey in Australia: introduction of a new tax is supported by three quarters of the respondents; but less preferred option than e.g. food labelling, advertising bans, mass media campaigns.
Feasibility	
Is the intervention feasible to implement?	
JUDGEMENT	RESEARCH EVIDENCE
No Probably no Probably yes Yes ***Varies*** Don't know	Systematic review concluded that federal junk food tax appears feasible based on product categories, but political feasibility was uncertain in 2018. Stakeholder-survey in the Netherlands: SSB tax is seen as an unpopular decision and efforts to adopt this policy may be counteracted by a strong lobby. SSB taxes have already been implemented in 12 European countries.

*Note*: For each EtD criterion the judgements and the corresponding research evidence is summarized. The detailed description of the research evidence is reported in Supplementary table S5. BMI, body mass index; EtD, Evidence to Decision; NCDs, non-communicable diseases; SSB, sugar-sweetened beverages.

#### The problem

The global burden of NCDs is significant. In 2019, NCDs such as cardiovascular diseases, cancer and type 2 diabetes accounted for 1.6 billion disability-adjusted life-years, and 42 million deaths worldwide.^13^ With obesity-mediated and direct effects on chronic diseases, the consumption of SSBs is strongly associated with NCD-related morbidity.^14^ SSBs are regularly consumed in Western Europe with an average consumption of 130 ml per day.^15^

#### Desirable and undesirable effects

Taxing SSBs might be an effective fiscal policy to decrease the purchase and consumption of SSBs, and to reduce the prevalence of poor health outcomes. A meta-analysis showed that SSB taxation reduces SSB consumption, with higher prices being associated with greater reductions. The evidence from six included studies showed that higher SSB prices may also decrease body mass index, and the prevalence of overweight and obesity.^16^ In another meta-analysis, the equivalent of a 10% SSB tax was associated with an average decline in beverage purchase and dietary intake of SSB of 10%.^17^ SSB taxation appears to be particularly effective in decreasing sales and consumptions, and the prevalence of overweight and obesity, when the tax is high, specific for beverage volume and applied to a broad definition of SSBs.^18^

Undesirable effects of SSB taxation were not investigated in any of the identified systematic reviews.

As a result of adaptation processes, a shift in consumption may be expected to offset the anticipated benefits. On a consumer’s level, individuals may compensate less purchase/intake of SSBs by consuming other unhealthy drinks or foods. Nevertheless, findings from individual studies are inconsistent about the presence of this shift.^19–21^ On a manufacturer’s level, beverages may only be reformulated by replacing sugar with non-nutritive sweeteners or other ingredients. However, the evidence on the health impact of non-nutritive sweeteners is conflicting.^22^^,^^23^ Another adaptation may be changes in marketing strategies and decreasing product size, with consumers paying more, but consuming the same amount of sugar.^6^

#### Certainty of the evidence

A systematic review by Afshin *et al*.^24^ investigated the impact of SSB pricing on dietary intake and obesity. The authors rated the certainty of evidence and net benefit of lower SSB consumption as moderate, whereas the rating for the net benefit reduction in body mass index was low.^24^

No other systematic review is available that reports the outcome-specific certainty of evidence of an SSB tax. An ongoing Cochrane review investigates the effect of SSB taxation for preventing obesity, but the results are not available yet.^25^ A web-based survey of guideline panels showed that a high certainty of evidence was the strongest predictor for a strong recommendation.^26^

#### Values

No evidence from systematic reviews was identified to determine societal values regarding outcomes that result from SSB tax for prevention of obesity. However, NCDs represent the main causes of disability and mortality worldwide, and obesity is a key underlying risk factor. Improvements in these health-related outcomes are thus important on a population level.^13^^,^^27^ Moreover, reducing risk of obesity and its consequences are of public interest since they result in lower costs for health services and social welfare systems.^28^

#### Balance of effects

There is low to moderate certainty of evidence that SSB pricing is able to reduce the purchase and consumption of SSBs and thus may have a positive effect on health-related outcomes such as obesity.^24^ Reducing obesity and reducing the burden of NCDs are outcomes of priority for the population of the country at hand. Adverse effects are currently considered small, but varied. Of note, they have been poorly reported in the literature so far. The balance between desirable and undesirable effects probably favours recommending an SSB tax versus not recommending an SSB tax.

#### Resources required (or savings) and cost-effectiveness

In a cost-effectiveness analysis of a US National SSB tax, the implementation of a penny-per-ounce SSB tax was highly cost-saving (24 times the tax implementation costs). In this modelling study, SSB tax generated high tax revenues and health cost savings for the government, individuals and the private sector. For the beverage industry, net costs varied depending on the scenario of how tax was passed on to consumers, from $0.9 billion to $49.8 billion.^29^ A modelling study in Canada on the effects of a 20% SSB tax estimated an annual levy of $30 to $35 per person, resulting in an overall tax burden of about $1.1 billion. The estimated direct healthcare saving ranged from $1.7 to 2.0 billion per quintile lifetime, depending on the income group.^30^

#### Equity

Individuals with a lower socioeconomic status are at risk of consuming more SSBs,^31^ as well as of suffering from NCDs more frequently.^32^ A systematic review found evidence that increasing the price of SSBs impacts health in a positive manner, and more so among individuals with a low than among those with higher socioeconomic status; it may therefore contribute to a reduction of health inequalities.^33^ Another systematic review evaluated distributional equity in studies on various taxes on unhealthy commodities and found that evidence on equity is generally poorly described. Results of existing evaluations of SSB taxation were inconsistent and varied across income groups.^34^

#### Acceptability

A mixed-method systematic review and meta-analysis published on behalf of the PEN consortium investigated the political and public acceptability of an SSB tax in various countries. Pooled proportions indicated that 39–66% of the general public support SSB taxation. The authors, however, were not able to estimate political acceptability, given that no quantitative studies on political acceptability fulfilled the inclusion criteria.^35^

#### Feasibility

SSB taxation is a frequently used political strategy: to date, taxes on SSBs have already been implemented in 50 countries, among them 12 European countries.^36^ A technical paper, commissioned by the WHO, summarizes food taxes implemented in several countries and reports that common challenges in implementation are a lack of capacity in tax administration, and poor monitoring and evaluation of health impacts. Moreover, taxes are often set at too low levels to be effective in influencing consumers’ behaviour.^37^

### Drawing conclusions

**Figure 2:**
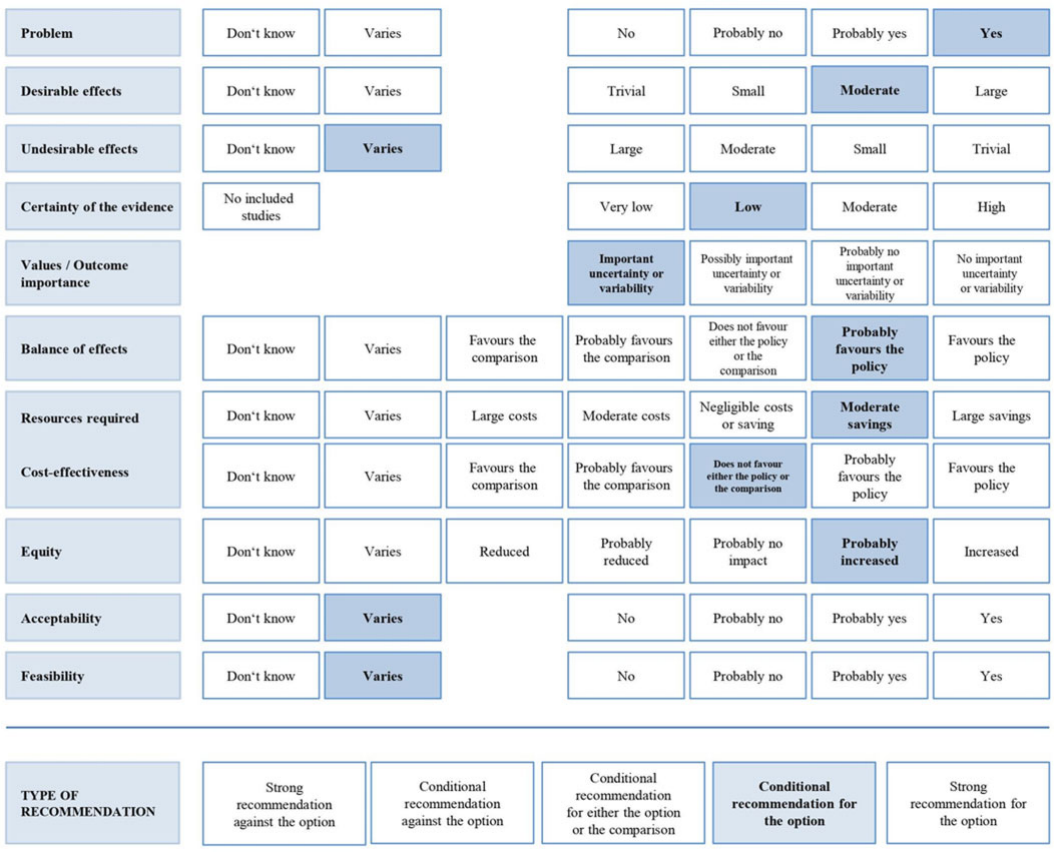
Panel’s judgements in relation to each EtD criterion, and strength of recommendation after considering all criteria. Judgements by the panel are highlighted.

The evidence summarized by the technical team was discussed by the panel. Figure 2 shows the summary of judgements regarding a tax on SSBs for the prevention of obesity based on the EtD frameworks. As a result of the high priority to prevent and reduce the burden of obesity and because of the large desirable effects as well as considering all other EtD criteria, the panel voted in favour of the recommendation. The panel finally made a conditional recommendation for the implementation of an SSB tax, since high-certainty evidence was missing for the criteria ‘undesirable effects’, ‘acceptability’ and ‘feasibility’ of the policy and judgements were often based on indirect evidence (e.g. SSB taxation of 10% taxation instead of 20%). The panel suggested generating more evidence with regards to the undesirable effects, acceptability and feasibility of the policy, and the evaluation of the specific design of taxation (20% taxation on SSBs based on their sugar density).

Based on the EtD tables and the panel’s recommendation, decision-makers may decide to conduct a pilot study prior to implementing the policy at a national level. For instance, an SSB tax may be implemented in some geographical areas (e.g. only in specific counties or states) and evaluated.

## Discussion

We illustrated the application of the GRADE EtD frameworks in the area of health policy-making by developing a fictitious recommendation on SSB taxation. We took the role of a fictitious guideline panel, provided exemplary evidence for each EtD criterion, drew conclusions and finally developed a fictitious recommendation. By using the GRADE EtD frameworks, we were able to show that guideline panels can structure their process of making recommendations in the field of health nutrition polices and make their use of evidence systematic and transparent. Elaborated EtD tables and recommendations can be used by policy-makers and facilitate evidence-informed discussions and decision-making in healthcare and public health.

Within the EtD framework process, guideline panels formulate strong or conditional recommendations to support policy-making. Policy-makers, however, need to act upon them in a ‘black-and-white’ manner. For them, the available options are: not implementing the policy, postponing the decision, conducting a pilot study prior to fully implementing, implementing with an impact evaluation, or fully implementing.^5^

Another aspect to consider is the observation that policy-makers are more likely to understand the summarized evidence presented in an EtD framework than to read and understand a full set of systematic reviews.^38^ However, it is important that panel members are familiar with the EtD frameworks and their role in policy-making. Their usage can be meaningful and ensure a transparent and systematic approach when introducing health policies. Furthermore, the approach strengthens the position of health topics in the political area and can help to strengthen the position vis-à-vis other topics. A survey has recently shown that GRADE is not used regularly for actual policy-making in Europe.^9^ Overall, high-quality EtD frameworks require methodological expertise and resources; both are often in short supply in (guideline) organizations.

A limitation of our fictitious example is the potential simplification of a health policy on SSB taxation. We assumed a mostly linear pathway of effects, i.e. that an increased SSB price would decrease the consumption of SSBs and that this would in turn decrease the risk of obesity and therefore increase overall population health and wellbeing. Taking a complexity perspective into account, the introduction of the tax in this example may lead to reformulation of SSBs, changes in marketing strategies by industry actors, diversification of product ranges, and price changes in products other than SSBs. Furthermore, the social discourses and media attention on the health effects of SSBs arising from the political debate on the introduction of an SSB tax in a given context can affect SSB consumption independently of the price increase.^39^ Therefore, our example needs to be interpreted with caution.

Another limitation is that we chose a fictitious example and provide summaries of exemplary evidence and judgements. Therefore, it is possible that not all available evidence for the EtD criteria was adequately considered when formulating our recommendation. Performing a real trustworthy guideline on the health impacts of SSB taxation requires high-quality reviews for the EtD criteria. For several EtD criteria, however, there was only evidence from single studies or indirect evidence and therefore several assumptions had to be made. For example, no systematic review was available on the unintended or adverse effects of SSB taxation. Overall, no systematic review focused exclusively on SSB taxation with a 20% rate and a threshold of 5 g of sugar per 100 ml of drinks. Therefore, we relied on individual studies. In the course of a real guideline process, the technical team would ideally conduct *de novo* a full systematic or scoping review for each EtD criterion, or rely on existing high-quality systematic reviews.

Finally, additional decision criteria beyond those included in the EtD framework we prepared here - such as existing policies and environmental outcomes - may be of relevance in health policy-making, notably in the context of a Health in All Policies approach. We did not explicitly consider them in our fictitious example application but they could be added as separate criteria or included under existing EtD criteria when applying the GRADE EtD frameworks or when using an alternative EtD framework, such as the WHO-INTEGRATE framework^40^ if deemed critical for decision-making.

Further it is important to note that an SSB tax is only one of several potential actions to reduce the burden of obesity and associated NCDs.^10^ Since obesity is a multifactorial disease an isolated intervention may only have marginal impacts on public health. Health policies might therefore incorporate multiple policy actions (e.g. in the field of nutrition, physical activity or sedentary behaviour), and also address health inequities (e.g. in education, infrastructure), to be maximally effective in the reduction of poor health outcomes.^18^ However, the evidence presented in this article suggests that a tax on SSBs may be an effective component in future public health policies.

We illustrated the process of developing a fictitious recommendation for an SSB tax for the prevention of obesity, showing that the GRADE EtD frameworks can be used to support the process of health policy-making. GRADE EtD frameworks can help guideline panels by structuring their discussion, adding transparency to the process, and helping to make the most of available evidence while taking into consideration different important viewpoints.

## Supplementary data

Supplementary data are available at *EURPUB* online.

## Supplementary Material

ckac077_Supplementary_Data
