# Supplementary material for: Using GRADE Evidence to Decision frameworks to support the process of health policy-making: an example application regarding taxation of sugar-sweetened beverages
Source: Eur J Public Health. 2022 Nov 29;32(Suppl 4):iv92–iv100. doi: 10.1093/eurpub/ckac077 (PMC9706117; doi:10.1093/eurpub/ckac077)
Supplement: ckac077_Supplementary_Data [file ckac077_supplementary_data.zip › ckac077_Supplementary_Data/Stadelmaier_UsingGradeEvidenceToDecisionFrameworks_SupplMat_marked.pdf]

Supplementary Material

Using GRADE Evidence to Decision frameworks to support the process of health policy-making: an example application regarding taxation of sugar-sweetened beverages

Julia Stadelmaier[1], Eva A Rehfuess[2,3], Sarah Forberger[4], Angelika Eisele-Metzger[1,5], Blin Nagavci[1], Holger J Schünemann[6], Joerg J Meerpohl[1,5], Lukas Schwingshackl[1] on behalf of the PEN consortium

[1] Institute for Evidence in Medicine, Medical Centre - University of Freiburg, Faculty of Medicine, University of Freiburg, Freiburg, Germany.

[2] Institute for Medical Information Processing, Biometry and Epidemiology (IBE), LMU Munich, Munich, Germany

[3] Pettenkofer School of Public Health, Munich, Germany

[4] Department of Prevention and Evaluation, Leibniz-Institute for Prevention Research and Epidemiology – BIPS, Bremen, Germany

[5] Cochrane Germany, Cochrane Germany Foundation, Freiburg, Germany

[6] Department of Health Research Methods, Evidence and Impact, Department of Medicine, McMaster University, Hamilton, Canada

Supplementary Table 1: Overview of guidelines using GRADE Evidence to Decision frameworks in the process of developing recommendations (adapted from Zähringer et al.[1])

WHO Guideline	Recommendations	Strength of recommendations	Notes on using GRADE EtD frameworks
Potassium intake for adults and children[2]	Increase in potassium intake from food for reduction of blood pressure and risk of cardiovascular disease, stroke and coronary heart disease in adults<br><br>Potassium intake of at least 90 mmol/day (3510 mg/day) for adults<br><br>Increase in potassium intake from food to control blood pressure in children	Strong<br><br>Conditional<br><br>Conditional	"*GRADE methodology was followed to prepare evidence profiles related to preselected topics, based on up-to-date systematic reviews of the scientific literature.*"<br><br>Criteria used: (i) Certainty of evidence, (ii) values and preferences, (iii) trade-off between benefits and harm, (iv) costs and feasibility
Sodium intake for adults and children[3]	Reduction in sodium intake to reduce blood pressure and risk of cardiovascular disease, stroke and coronary heart disease in adults<br><br>Reduction to <2 g/day sodium (5 g/day salt) in adults<br><br>Reduction in sodium intake to control blood pressure in children. The recommended maximum level of intake of 2 g/day sodium in adults should be adjusted downward based on the energy requirements of children relative to those of adults.	Strong<br><br>Strong<br><br>Strong	"*The systematic reviews and the GRADE evidence profiles for each of the critical outcomes were used for drafting the guideline.*"<br><br>Criteria used: (i) Certainty of evidence, (ii) values and preferences, (iii) trade-off between benefits and harm, (iv) costs and feasibility
Sugar intake for adults and children[4]	Reduced intake of free sugars throughout the life course.<br><br>Reducing the intake of free sugars to less than 10% of total energy intake in both adults and children.<br><br>Further reduction of the intake of free sugars to below 5% of total energy intake.	Strong<br><br>Strong<br><br>Conditional	"*The systematic reviews and the GRADE evidence profiles for each of the critical outcomes were used for drafting the recommendations.*"<br><br>"*The GRADE evidence profiles incorporated the discussions and inputs from the NUGAG Subgroup on Diet and Health, based on the outcomes of the systematic reviews.*"

			Criteria used: (i) Certainty of evidence, (ii) values and preferences, (iii) trade-off between benefits and harm, (iv) costs and feasibility
Assessing and managing children at primary health-care facilities to prevent overweight and obesity in the context of the double burden of malnutrition[5]	Not providing supplementary foods routinely to moderately wasted infants and children (i.e. with acute undernutrition)<br><br>Not providing supplementary foods for treating stunting (chronic undernutrition) among infants and children<br><br>Health workers should provide nutrition counselling to caregivers of overweight children aged less than 5 years<br><br>Health workers should provide counselling on physical activity to caregivers of overweight children aged less than 5 years<br><br>Children aged less than 5 years who are identified as obese should be assessed and an appropriate management plan should be developed. This can be done by a health worker at primary health-care level, if adequately trained, or at a referral clinic or local hospital	Conditional<br><br>Conditional<br><br>Conditional<br><br>Conditional<br><br>Conditional	"*GRADE methodology was followed, to prepare evidence profiles related to pre-selected topics, based on up-to-date systematic reviews.*"<br><br>"*The GRADE tables were prepared using the GRADE profiler software (GRADEPro), when appropriate.*"<br><br>Criteria used: (i) Benefits and harms, (ii) certainty of evidence, (iii) values, preferences and acceptability, (iv) feasibility and resource use, (v) equity.

GRADE: Grading of Recommendations Assessment, Development and Evaluation; NUGAG: WHO Nutrition Guidance Expert Advisory Group; WHO: World Health Organisation

Supplementary Table 2: Criteria for Evidence to Decision frameworks for policy-making and evaluation (adapted from Moberg et al.[6])

**Priority of the problem**	*Is the problem a priority?*<br>Prioritisation of health problems is influenced for example by the prevalence, incidence and consequences of the disease, but also by national or international health targets.
**Benefits and harms**	*How substantial are the desirable anticipated effects?*<br>*How substantial are the undesirable anticipated effects?*<br>For each health outcome, beneficial and harming effects of the health policy are gathered.
**Certainty of the evidence**	*What is the overall certainty of the evidence of effects?*<br>The certainty of the evidence across a body of evidence is assessed using the GRADE[7] approach.
**Values**	*Is there important uncertainty about or variability in how much people value the main outcomes?*<br>Outcomes are prioritised in terms of their importance to the population, and the certainty of this importance.
**Balance of effects**	*Does the balance between desirable and undesirable effects favour the intervention or the comparison?*<br>Panels compare both desirable and undesirable effects, and judge whether the balance favours a decision for or against the recommendation of a health policy.
**Resources required and cost-effectiveness**	*How large are the resource requirements (costs)?*<br>*What is the certainty of the evidence of resource requirements (costs)?*<br>*Does the cost-effectiveness of the intervention favour the intervention or the comparison?*<br>Panels consider the resource use (or savings) and the cost-effectiveness of the policy under discussion and compare it to alternative options.
**Equity**	*What would be the impact on health equity?*<br>The impact of the policy on health equity is discussed.
**Acceptability**	*Is the intervention acceptable to key stakeholders?*<br>Panels examine whether recommending a policy may be supported by policy-makers, special interest groups and the general public.
**Feasibility**	*Is the intervention feasible to implement?*<br>Barriers and facilitators in implementing the health policy are discussed.

Supplementary Appendix 1:

Methodological guidance publications and other literature resources

The GRADE Working Group has published numerous methodological guidance papers, mainly in the Journal of Clinical Epidemiology.[8-11] For the present work, we build on these publications, as well as on the guidance for GRADE EtD frameworks for clinical recommendations published in the British Medical Journal by Alonso-Coello and colleagues,[12 13] and its adaption for health system and public health decisions by Moberg and colleagues.[6]

Furthermore, we used findings of our scoping review on the use of GRADE in the field of nutrition and physical activity policy-making and evaluation. Our scoping review showed that the GRADE approach is, in principle, able to support the decision-making process of health policy-making and evaluation through facilitating a structured and transparent use of evidence.[1]

Supplementary Table 3: Summary of panel members' disclosure of interests

Panel member	Role	Financial interests	Non-financial interests[a]	Actions taken[b]
Panel member 1	Chair of panel; voting panel member; methodologist	None	None	None
Panel member 2	Guideline methods editor; voting panel member; methodologist	None	None	None
Panel member 3	Voting panel member; epidemiologist	None	None	None
Panel member 4	Voting panel member; public health scientist	None	None	None
Panel member 5	Voting panel member; nutrition scientist	None	None	None
Panel member 6	Voting panel member; physician	None	None	None
Panel member 7	Voting panel member; bioinformatician	None	None	None
Panel member 8	Voting panel member; economist	None	None	None
Panel member 9	Voting panel member; economist	None	None	None
Panel member 10	Voting panel member; sociologist	None	None	None
Panel member 11	Voting panel member; ethicist	None	None	None
Panel member 12	Voting panel member; physician	None	None	None

[a] Non-financial interests arise, for instance, from a competing professional, academic, personal or political role.
[b] Traversy and colleagues[14] provide a practical guidance for managing conflicts of interest in the development of health guidelines.

Supplementary Table 4: Detailed description of the guideline question.

QUESTION

**Should SSB taxation vs. no taxation be used for health outcomes?**	
**PROBLEM:**	Overweight and obesity are growing health concerns in the exemplary country. About half of the adult population is overweight, or even obese. The resulting health consequences are significant, since obesity is one major risk factor for the development of NCDs. The consumption of SSB is strongly associated with the morbidity of NCDs. The average daily intake of SSB in the exemplary country is high.
**OPTION:**	Taxation on SSB: Every non-alcoholic drink with added sugar is liable for the levy. These are for example sodas, energy or sports drink, or sugar-sweetened tea. The amount of taxation is determined by the energy density (grams of sugar per 100 ml of drink). The tax will be applied with a rate of 20% for drinks with more than five grams of sugar per 100 ml.
**COMPARISON:**	No taxation on SSB.
**MAIN OUTCOMES:**	Anthropometric outcomes, e.g. risk of overweight (BMI: ≥25-29.9 kg/m²), obesity (BMI: ≥30 kg/m²), change in body weight (kg), fat mass (kg), waist circumference (cm), BMI (kg/m²).<br>NCDs, e.g. cardiovascular diseases such as coronary heart disease and stroke; type 2 diabetes; and cancer.<br>Dietary behaviour outcomes, e.g. intake of sugar-sweetened beverages (ml/d), total energy consumed (kcal/d)<br>Economic parameters, e.g. implementation costs, tax revenues ($).
**SETTING:**	European high-income country
**PERSPECTIVE:**	General population
**BACKGROUND:**	For the nutrition association of the exemplary country, reducing obesity and its associated consequences are a priority. Moreover, obesity prevention is crucial for achieving the sustainable development goal target 3.4 to reduce by one-third premature mortality from NCDs by 2030.
**CONFLICT OF INTERESTS:**	No conflict of interest.

BMI: Body Mass Index; EtD: Evidence to Decision; NCDs: non-communicable diseases; SSB: sugar-sweetened beverages;

Supplementary Table 5: Detailed description of the GRADE Evidence to Decision (EtD) criteria for the fictitious sugar-sweetened beverage taxation in the prevention of obesity and its associated non-communicable diseases. For each EtD criteria the judgments and the corresponding research evidence are summarised.

**Problem**<br><br>Is the problem a priority?		
**JUDGEMENT**	**RESEARCH EVIDENCE**	**ADDITIONAL CONSIDERATIONS**
○ No<br>○ Probably no<br>○ Probably yes<br>● Yes<br>○ Varies<br>○ Don't know	The global burden of NCDs is significant. In 2019, NCDs such as cardiovascular diseases, cancer, chronic respiratory diseases, and type 2 diabetes accounted for 1.6 billion DALYs and 42 million deaths worldwide.[15] Obesity, hypertension, dyslipidaemia, and high blood glucose are among the major risk factors for the development of NCDs.[16]<br><br>Overweight and obesity are growing health concerns in European countries. In our exemplary country, about half of the adult population is overweight (BMI ≥25–29.9 kg/m^2), and one fifth is even obese (BMI ≥30 kg/m^2).[17] Among children and adolescents, the prevalence of overweight and obesity are 15% and 6%, respectively.[18]<br><br>With obesity-mediated and direct effects on chronic diseases, the consumption of SSB is strongly associated with NCD-related morbidity. According to a modelling study by the Global Burden of Disease study group, more than 8.5 million DALYs and 180.000 deaths worldwide were linked to SSB intake. Among these, more than 0.2 million DALYs and 11.400 death occur in Western Europe, and more than 64.000 DALYs and 3.000 deaths in our selected corresponding country, respectively. The study also highlights sociodemographic variability, with more than 70% of SSB-related deaths occurring in middle-income countries and the highest proportional burden among younger adults (20-44 years).[19] A recent comparative risk assessment study evaluated the impact of 12 food groups including SSB on DALYs from different NCDs, across 16 European countries. It was shown that a high consumption of SSB had significant health impacts for coronary heart disease and type 2 diabetes.[20]<br><br>SSB are regularly consumed worldwide, with a global average of about 200 ml per day (ml/d) in adults. Consumption varies by country, sex and age group. In Western Europe, the average is at 130 ml/d, with the highest consumption in young adults under the age of 40 years (220 ml/d in women and 250 ml/d in men).[21] In children and adolescents a trend of increasing consumption of SSB can be observed from 1990 to 2000, which has been stabilised or gradual declined from 2000 to 2010.[22] In Europe, it is estimated that adolescents consume about 228 ml/d, which makes SSB the second most consumed fluid after water. In a comparison including eight countries, adolescents of our corresponding country consumed the highest amount of energy from beverages per capita, with more than 200 kcal coming from the consumption of SSB.[23]	None

Desirable Effects<br><br>How substantial are the desirable anticipated effects?		
**JUDGEMENT**	**RESEARCH EVIDENCE**	**ADDITIONAL CONSIDERATIONS**
○ Trivial<br>○ Small<br>● Moderate<br>○ Large<br>○ Varies<br>○ Don't know	Taxing SSB might be an effective fiscal policy to decrease the purchase and consumption of SSB, and to reduce the prevalence of poor health outcomes: A meta-analysis showed that SSB tax reduces SSB consumption, with higher prices being associated with greater reductions. The evidence from six included studies showed that higher SSB prices may also decrease BMI, and the prevalence of overweight and obesity.[24] In a systematic review by Teng and colleagues[25], the equivalent of a 10% SSB tax was associated with an average decline in beverage purchases and dietary intake of SSB of 10%. According to the systematic review by Itria and colleagues[26], SSB tax appears to be particularly effective in decreasing sales and consumptions, and the prevalence of overweight and obesity, when the tax is high, specific for beverage volume, and applied to a broad definition of SSB. However, changes in sales seem to be small in high-income countries.<br><br>In a Cochrane Review on environmental interventions to reduce the consumption of SSB and their effects on health, the authors showed that evidence indicates that SSB price increases were effective and showed no harmful effects.[27]<br><br>In 2015, the WHO published a guideline on sugar intakes in children and adults. The guideline recommends for both adults and children, to reduce the intake of free sugars to less than 10% of total energy intake (strong recommendation), even though the certainty of evidence was not judged as high, but mostly as moderate or low.[4] National health societies of our exemplary country provide similar recommendations and set the level of maximum daily intake of free sugars at 50 g (based on an estimated total energy intake of 2,000 kcal/d).[28]	Direct evidence that focus specifically on the effects of a 20% taxation is not available.<br><br>Effects on NCDs are difficult to assess, since long-term studies are often not feasible.

Undesirable Effects<br><br>How substantial are the undesirable anticipated effects?		
**JUDGEMENT**	**RESEARCH EVIDENCE**	**ADDITIONAL CONSIDERATIONS**
○ Large<br>○ Moderate<br>○ Small<br>○ Trivial<br>● Varies<br>○ Don't know	No systematic review is available on the unintended or adverse effects of SSB taxation.<br><br>As a result of adaption processes, a shift in consumption may be expected to offset the anticipated benefits. On a consumer's level, individuals may compensate less purchase / intake of SSB by consuming other unhealthy drinks of foods. Nevertheless, finding from individual studies are inconsistent about the presence of this shift: Findings of a randomised controlled trial on grocery shopping in a virtual supermarket showed that SSB tax decreased SSB purchase, however, had no impact on the total energy content of the total weekly shopping cart.[29] An evaluation one year after the introduction of the UK soft drinks industry levy reports no change in the purchase of soft drinks, but the amount of sugar consumed per household decreased by 30 g (10%).[30] In Philadelphia, the SSB tax of 1.5 cents/ 28.5 ml revealed no	See Additional considerations above.

changes in purchase of snacks and alcohol, but an increase in the purchase of beverage concentrates.[31] A modelling study based on evaluations of the SSB taxes implemented in France, a shift was observed with a decrease in SSB sales, but increase of overall soft drink sales.[32]

On a manufacturer's level, beverages may only be reformulated by replacing sugar with non-nutritive sweeteners or other ingredients. However, the evidence on the health impact of non-nutritive sweeteners is conflicting.[33 34] Another adaptation may be changes in marketing strategies and decreasing product size, with consumers paying more, but consuming same amounts of sugar.[35]

A loss of regional jobs is a major industry argument against SSB taxation. In a modelling study, Powell and colleagues[36] indicate that the introduction of a 20% SSB tax does not change the employment situation in two US states. By shifting consumers' spending, and reinvesting the revenue from the SSB tax, total employment in the SSB industry may actually increase, and the decline in employment in the SSB industry may be offset by the creation of new jobs in the non-SSB industry.

Certainty of evidence

What is the overall certainty of the evidence of effects?

JUDGEMENT

- ○ Very low
- ● Low
- ○ Moderate
- ○ High
- ○ No included studies

RESEARCH EVIDENCE

Outcomes	Plain language statements	Absolute Effect: With no taxation	Absolute Effect: With Sugar sweetened beverage taxation	Absolute Effect: Difference	Relative effect (95% CI)	Certainty of the evidence GRADE
**Body Mass Index (observational studies)** Follow-up: mean 51,160 patient years	*The net benefit reduction in Body Mass Index was small and the certainty of evidence was rated as low.*	18.46 kg/m2	18.4 kg/m2	Average difference (MD): 0.06 kg/m2 lower (95% CI: 0.16 kg/m2 lower to 0.03 kg/m2 higher) Based on data from 30180 patients in 2 studies	-	⊕⊕◯◯ LOW
**SSB intake (observational studies: before-and after studies)** Follow-up: range 1 months to 9	*The net benefit reduction in SSB intake was moderate and the certainty of evidence was rated as moderate.*	120 ml/d	111.14 ml/d	Average difference (MD): 8.86 ml/d lower (95% CI: 11.43 to 6.3 ml/d lower Based on data from 30 patients in 2 studies	-	⊕⊕⊕◯ MODERATE Due to serious risk of bias.
**SSB intake (observational studies: cohort studies)** Follow-up: range 48 months to 240	*The net benefit reduction in SSB intake was moderate and the certainty of evidence was rated as low.*	120 ml/d	115.55 ml/d	Average difference (MD): 4.45 ml/d lower (95% CI: 9.24 ml/d lower to 0.33 ml/d higher Based on data from 32406 patients in 2 studies	-	⊕⊕◯◯ LOW

ADDITIONAL CONSIDERATIONS

U.S. Preventive Services Task Force grading system[47] was used to rate the certainty of the evidence.

A systematic review by Asfhin and colleagues[37] investigated the impact of SSB pricing on dietary intake and obesity. By using the U.S. Preventive Services Task Force grading system the authors rated the certainty of evidence and net benefit of lower SSB consumption as moderate, whereas the rating for the net benefit reduction in BMI was low.[37]

No other systematic review is available reporting the outcome-specific certainty of evidence of an SSB tax. A recent Cochrane review investigated the health effects of taxation of sugar or sugar-added foods in the general population. Based on the body of evidence from one modelling study, the authors reported very low certainty of the evidence for a reduced consumption of taxed sugar-added foods.[38] An ongoing Cochrane review investigates the effect of taxation of SSB for preventing obesity, but the results are not available yet.[39]

Indirect evidence is available from a systematic review commissioned by the WHO, which showed that a reduction in free sugars in adults and children was probably beneficial for improving anthropometric outcomes such as body weight (moderate certainty of evidence). Moreover, the authors rated the certainty of evidence for a positive association between high intake of free sugars and risk of overweight in children as low.[40]

In a series of dose-response meta-analyses of prospective observational studies, each daily serving increase in SSB (250 ml/d) was positively associated with risk of type 2 diabetes (high certainty of evidence),[41] all-cause mortality (low certainty of evidence),[42] cardiovascular disease (moderate certainty of evidence),[43] colorectal cancer (low certainty of evidence),[44] hypertension (low certainty of evidence),[45] and adiposity (low certainty of evidence)[46].

Values

Is there important uncertainty about or variability in how much people value the main outcomes?

JUDGEMENT

○ Important uncertainty or variability
○ Possibly important uncertainty or variability
○ Probably no important uncertainty or variability
● No important uncertainty or variability

RESEARCH EVIDENCE

Outcomes	Importance	Certainty of the evidence (GRADE)
BMI (observational studies)<br>assessed with: kg/m2<br>follow-up: mean 51,160 patient years	Critical	⊕⊕◯◯<br>Low
SSB intake (observational studies: before- and after studies)<br>assessed with: ml/d<br>follow-up: range 1 months to 9	Critical	⊕⊕⊕◯<br>Moderate
SSB intake (observational studies: cohort studies)<br>follow-up: range 48 months to 240	Critical	⊕⊕◯◯<br>Low

No evidence from systematic reviews was identified to determine societal values regarding outcomes that result from

ADDITIONAL CONSIDERATIONS

None.

	SSB tax for prevention of obesity. However, weight loss is reported to be an important outcome, as according to the Centres for Disease Control and Prevention almost 50% of adults in the US tried to reduce body weight in the previous 12 months.[48] Furthermore, NCDs such as coronary heart disease and stroke represent the main causes of disability and mortality worldwide, and obesity is a key underlying risk factor. Improvements in these health-related outcomes are thus an important outcome on a population level.[15] This is in line with the WHO guideline on sugar intake from 2015, which places a high value on reduction of risk of overweight, obesity and associated NCDs, and highlighted that interventions to reduce the burden of NCDs are valuable.[4]<br><br>Moreover, NCDs and their associated disabilities have a considerable impact not only on a person's daily life, but also on that of their close social network such as family and friends, and they contribute to a higher burden for their caregivers.[49] Finally, reducing risk of obesity and its consequences are of public interest since they result in lower costs for health services and social welfare systems. It has been estimated that obesity accounts for 0.7-2.8% of a country's total health-care costs, and that direct medical costs of obese individuals are 30% higher than that of their normal-weight peers.[50] In our corresponding country, an estimated 100,000 individuals die prematurely each year due to obesity, and there is a marked surplus of unemployment, long-term nursing care, and burden (e.g. from comorbidities, but also stigmatization, prejudices, etc.).[51]<br><br>There is no reason to believe there is important uncertainty about or variability in how much people value reducing the risk of obesity and NCDs.	
**Balance of effects**<br><br>Does the balance between desirable and undesirable effects favour the intervention or the comparison?		
**JUDGEMENT**	**RESEARCH EVIDENCE**	**ADDITIONAL CONSIDERATIONS**
○ Favours the comparison<br>○ Probably favours the comparison<br>○ Does not favour either the intervention or the comparison<br>● Probably favours the intervention<br>○ Favours the intervention<br>○ Varies<br>○ Don't know	There is low to moderate certainty of evidence that SSB pricing is able to reduce the purchase and consumption of SSB and thus may have a positive effect on health-related outcomes such as obesity.[37] Reducing obesity and reducing the burden of NCDs are outcomes of priority for the population of the country at hand.<br><br>Adverse effects are currently considered small, but varied. Of note, they have been poorly reported in the literature so far. Adaptation processes, such as a shift in individual's consumptions towards other unhealthy foods and reformulation or marketing strategies by the manufacturers, are to be expected and may offset the anticipated effects. Moreover, a modelling study points at a possibly low sustainability effect. Based on evaluations of the SSB taxes implemented in France and Hungary, only small effects on the SSB sales were shown, which disappeared after two years in the case of Hungary.[32]<br><br>The balance between desirable and undesirable effects probably favours the recommending an SSB tax versus not recommending an SSB tax.	See the four preceding criteria.

**Resources required**<br><br>How large are the resource requirements (costs)?		
**JUDGEMENT**	**RESEARCH EVIDENCE**	**ADDITIONAL CONSIDERATIONS**
○ Large costs<br>○ Moderate costs<br>○ Negligible costs and savings<br>● Moderate savings<br>○ Large savings<br>○ Varies<br>○ Don't know	The implementation of SSB results in additional taxes generated by the country: An analysis of implementing a 10% SSB tax in South Africa estimated a total tax revenue of $450 million per year.[52] In Canada, a modelling study on the effects of a 20% tax estimated an annual levy of $30 to $35 per person, resulting in overall revenue of about $1.1 billion.[53]<br><br>In the modelling study by Wilde and colleagues[54], a tax on SSB ($0.01 per ounce) generated high tax revenues. Net costs for the beverage industry varied depending on the scenario of how tax was passed on to consumers, from $0.92 billion (100% pass-through, largely attributable to tax-implementation costs), to $49.75 billion (50% pass-through, partial industry coverage).[54]	Estimated costs are not available for a European country.

**Certainty of evidence of required resources**<br><br>What is the certainty of the evidence of resource requirements (costs)?		
**JUDGEMENT**	**RESEARCH EVIDENCE**	**ADDITIONAL CONSIDERATIONS**
○ Very low<br>○ Low<br>○ Moderate<br>○ High<br>● No included studies	Not applicable	Not applicable.

**Cost effectiveness**<br><br>Does the cost-effectiveness of the intervention favour the intervention or the comparison?		
**JUDGEMENT**	**RESEARCH EVIDENCE**	**ADDITIONAL CONSIDERATIONS**
○ Favours the comparison<br>○ Probably favours the comparison<br>○ Does not favour either the intervention or the comparison<br>● Probably favours the intervention<br>○ Favours the intervention<br>○ Varies<br>○ No included studies	In a cost-effectiveness analysis of a National SSB tax in the US, Wilde and colleagues[54] evaluated health gains, taxes paid, and out-of-pocket health care savings for six distinct consumer categories, and showed that a penny-per-ounce SSB tax was highly health cost-saving (24 times the tax-implementation costs). In an analysis on implementing SSB taxation in South Africa, it was estimated that a 10% increase in the tax would prevent about 8,000 type 2 diabetes-related premature death over a period of 20 years, and generate cost-savings of about $140 million in subsidised health care and $450 million in tax revenue.[52] Kao and colleagues[53] evaluated the health care savings of a 20% tax on SSB in Canada. Their models estimated direct health care saving of $1.7 to $2.0 billion per quintile lifetime, depending on the income group.[53]<br><br>A microsimulation study in the US evaluated the cost-effectiveness of SSB tax with regard to their design and highlighted substantial health savings in absolute volume, sugar content tiers, or absolute sugar content taxes. For the two designs based on sugar content, however, health and economic benefits were about twice as large as volume based taxes.[55]	Estimated costs are not available for a European country.
**Equity**<br><br>What would be the impact on health equity?		
**JUDGEMENT**	**RESEARCH EVIDENCE**	**ADDITIONAL CONSIDERATIONS**
○ Reduced<br>○ Probably reduced<br>○ Probably no impact<br>● Probably increased<br>○ Increased<br>○ Varies<br>○ Don't know	Individuals with a lower socioeconomic status are at risk of consuming more SSB,[56] as well as of suffering from NCDs more frequently.[57] Backholer and colleagues[58] found evidence that increasing the price of SSB impacts health in a positive manner, and more so among individuals with a low socioeconomic status than among those with higher socioeconomic status; it may therefore contribute to a reduction of health inequalities. A systematic review by Jain and colleagues[59] evaluated distributional equity in studies on various taxes on unhealthy commodities and found that evidence on equity is generally poorly described. Results of existing evaluations of SSB taxation were inconsistent and varied across income-groups.[59]<br><br>In qualitative interviews conducted in the Netherlands, stakeholders point to the disproportional impact of SSB tax on people with a low socioeconomic status. As SSB are more often consumed in this socioeconomic group, purchase becomes more expensive, lowering the budgets of the households (in case they maintain their usual consumption). However, this impact on the financial situation may be compensated by health benefits.[60] Lal and colleagues[61] conducted a modelling study and provided reassurance that, under a broad range of plausible SSB tax effects, the	None.

	regressivity of the tax in terms of out-of-pocket costs to consumers would likely be exceeded by benefits in terms of averted costs in healthcare. In all tax scenarios, the lowest-socioeconomic groups are benefitting most from health gains.<br><br>Moreover, health equity can be promoted by allocating revenues generated by the SSB tax to projects that address the needs of disadvantaged groups or impacted communities.[55] A study among seven cities in the US showed that SSB tax have raised a total of $134 million annually, that were finally reinvested in early childhood programs, community improvements and increasing access to health foods and beverages.[62] Considering tax revenues for programs to increase health equity may provide an important counterpart to the price regressivity of SSB taxes.[55]	
**Acceptability**<br><br>Is the intervention acceptable to key stakeholders?		
**JUDGEMENT**	**RESEARCH EVIDENCE**	**ADDITIONAL CONSIDERATIONS**
○ No<br>○ Probably no<br>○ Probably yes<br>○ Yes<br>● Varies<br>○ Don't know	A mixed-method systematic review and meta-analysis published on behalf of the PEN consortium investigated the political and public acceptability of an SSB tax in various countries. Pooled proportions indicated that 39 to 66% of the general public support a tax on SSB, depending on question wording. The authors, however, were not able to estimate political acceptability of a tax on SSB, given that no quantitative studies on political acceptability fulfilled the inclusion criteria.[63]<br><br>In a recent survey conducted in the Netherlands, Eykelenboom and colleagues[64] assessed the public acceptability with regard to associated (e.g. sociodemographic) factors. The authors showed that SSB taxes tend to be supported more by people with a high socioeconomic level, low SSB consumption, normal weight status and no children at home. And, a tax on SSB was supported even more if its revenue is used for health initiatives. The political acceptability is addressed in an analysis of public testimonies in the Philadelphia City Council. The authors show that proponents of the tax emphasise the benefits of the revenue for infrastructure and education projects, with little mention of reducing consumption of SSB and improving health. In contrast, the arguments of the tax's opponents focus on the unfairness of targeting a single sector of the economy, on possible negative economic effects and on the supposed lack of evidence that the tax would actually influence consumer behaviour.[65]<br><br>A survey on Australian taxpayers provides evidence about the social acceptability of eight policy options (including SSB taxation) that aim to reduce obesity including SSB taxation. The authors report that the introduction of a new tax is supported by three quarters of the respondents, interpreting it as public desire to address the rising problem of obesity by a taxation policy. People that are most likely to benefit from the policy in terms of positive health outcomes are found to be the most willing to pay additional taxes. In comparison to the other options, SSB tax is less preferred than food labelling through traffic lights, advertising bans, improvements of food quality in public institutions and mass media campaigns.[66]	It may be favourable to inform the population of the concrete allocation of the additional revenues generated by the SSB tax.<br><br>Efforts to raise public awareness of obesity and NCDs, and the impact of SSB consumption may contribute to greater acceptance.

**Feasibility**<br><br>Is the intervention feasible to implement?		
**JUDGEMENT**	**RESEARCH EVIDENCE**	**ADDITIONAL CONSIDERATIONS**
○ No<br>○ Probably no<br>○ Probably yes<br>○ Yes<br>● Varies<br>○ Don't know	SSB taxation is a frequently used political strategy: To date, taxes on SSB have already been implemented in 50 countries, among them 12 European countries.[67] A technical paper, commissioned by the WHO, summarises food taxes implemented in several countries and reports that common challenges in implementation are the lack of capacity in tax administration, and poor monitoring and evaluation of health impacts. Moreover, taxes are often set at too low levels to be efficient in influencing consumers' behaviour.[68]<br><br>A recent systematic review investigated the legal and administrative feasibility of a federal junk food tax and concluded that federal junk food tax appears feasible based on product categories or combination category-plus-nutrient approaches. However, the authors concluded that political feasibility was uncertain in 2018 and seems unlikely to occur.[69] Perceived barriers in the implementation of SSB tax are addressed in a recent stakeholder-survey in the Netherlands. SSB tax is seen as an unpopular decision; efforts to adopt this policy may be counteracted by a strong lobby (i.e. food and beverage industry) and a lack of political will.[70]<br><br>With regard to the implementation of tobacco policies, George[71] summed up lessons learned that assist the implementation of a robust SSB tax. For example, it is reasonable to elaborate clearly the details of the tax and to tailor it to the countries objectives. Furthermore, the tax needs to be well communicated with its underlying rations (and evidence), to oppose the strong position of the industry. Moreover, the importance of monitoring and evaluation of the impacts is emphasised.	The technical paper provides information about the fiscal policies implemented in different countries worldwide with an overview of lessons learned.[68]

BMI: Body Mass Index; DALY: Disability-Adjusted Life-Year; EtD: Evidence to Decision; GRADE: Grading of Recommendations Assessment, Development and Evaluation; NCD: Non-Communicable Disease; PEN: Policy Evaluation Network; SSB: Sugar-Sweetened Beverages; UK: United Kingdom; US: United States; WHO: World Health Organisation

SUMMARY OF JUDGEMENTS

	JUDGEMENT						
PROBLEM	No	Probably no	Probably yes	**Yes**		Varies	Don't know
DESIRABLE EFFECTS	Trivial	Small	**Moderate**	Large		Varies	Don't know
UNDESIRABLE EFFECTS	Large	Moderate	Small	Trivial		**Varies**	Don't know
CERTAINTY OF EVIDENCE	Very low	**Low**	Moderate	High			No included studies
VALUES	Important uncertainty or variability	Possibly important uncertainty or variability	Probably no important uncertainty or variability	**No important uncertainty or variability**			
BALANCE OF EFFECTS	Favours the comparison	Probably favours the comparison	Does not favour either the intervention or the comparison	**Probably favours the intervention**	Favours the intervention	Varies	Don't know
RESOURCES REQUIRED	Large costs	Moderate costs	Negligible costs and savings	**Moderate savings**	Large savings	Varies	Don't know
CERTAINTY OF EVIDENCE OF REQUIRED RESOURCES	Very low	Low	Moderate	High			**No included studies**
COST EFFECTIVENESS	Favours the comparison	Probably favours the comparison	Does not favour either the intervention or the comparison	**Probably favours the intervention**	Favours the intervention	Varies	No included studies
EQUITY	Reduced	Probably reduced	Probably no impact	**Probably increased**	Increased	Varies	Don't know
ACCEPTABILITY	No	Probably no	Probably yes	Yes		**Varies**	Don't know
FEASIBILITY	No	Probably no	Probably yes	Yes		**Varies**	Don't know

TYPE OF RECOMMENDATION

Strong recommendation against the option	Conditional recommendation against the option	Conditional recommendation for either the option or the comparison	**Conditional recommendation for the option**	Strong recommendation for the option
○	○	○	●	○

CONCLUSIONS

Recommendation

The panel makes a conditional recommendation for the implementation of a tax on SSB.

Justification

Obesity and its associated NCDs are a health problem of priority for the country at hand. Implementing a tax on SSB may be a valuable and cost-saving option to reduce disability and poor health outcomes. However, there are not enough high-certainty evidence on the undesirable effects, and acceptability and feasibility in the context of the corresponding country.

Subgroup considerations

Not applicable.

Implementation considerations

Our recommendation and elaborated GRADE EtD tables may help policy-makers to decide on the implementation of a policy on SSB taxation. According to the GRADE EtD Frameworks for health system and public health decisions, they can chose between (1) not implementing the policy, (2) postponing the decision, (3) conducting a pilot study prior to fully implementing, (4) implementing the policy with an impact evaluation, (5) implementing the policy.

We suggest piloting the tax in a specific geographic area to generate more evidence with regard to the undesirable effects, acceptability and feasibility of the policy, and the evaluation of this specific design of taxation (20% taxation on SSB based on their energy density).

Monitoring and evaluation

Implementation of this recommendation should be subject to ongoing monitoring and evaluation to ensure high quality implementation adapted to the local context.

Research priorities

Explore the mechanism of effects behind this option.
More evidence with regards to the GRADE EtD criteria “undesirable effects”, “acceptability” and “feasibility” of SSB taxation is required.
More direct evidence is needed that focus on specific designs of SSB taxes, e.g. taxes based on energy density with a rate of 20%.

Supplementary References

1. Zähringer J, Schwingshackl L, Movsisyan A, et al. Use of the GRADE approach in health policymaking and evaluation: a scoping review of nutrition and physical activity policies. *Implement Sci* 2020;15(1):37. doi: 10.1186/s13012-020-00984-2

2. World Health Organisation. Guideline: Potassium Intake for Adults and Children. Geneva: World Health Organisation; 2012. [Available from: https://www.ncbi.nlm.nih.gov/books/NBK132453/?report=classic.

3. World Health Organisation. Guideline: Sodium Intake for Adults and Children. Geneva: World Health Organisation; 2012. [Available from: https://www.ncbi.nlm.nih.gov/books/NBK133309/.

4. World Health Organisation. Guideline: Sugars Intake for Adults and Children Geneva: World Health Organisation; 2015 [Available from: https://www.ncbi.nlm.nih.gov/pubmed/25905159.

5. World Health Organisation. Guideline: assessing and managing children at primary health-care facilities to prevent overweight and obesity in the context of the double burden of malnutrition. 2017.

6. Moberg J, Oxman AD, Rosenbaum S, et al. The GRADE Evidence to Decision (EtD) framework for health system and public health decisions. *Health Res Policy Syst* 2018;16(1):45. doi: 10.1186/s12961-018-0320-2

7. Hultcrantz M, Rind D, Akl EA, et al. The GRADE Working Group clarifies the construct of certainty of evidence. *J Clin Epidemiol* 2017;87:4-13. doi: 10.1016/j.jclinepi.2017.05.006

8. Guyatt GH, Oxman AD, Montori V, et al. GRADE guidelines: 5. Rating the quality of evidence—publication bias. *J Clin Epidemiol* 2011;64(12):1277-82. doi: 10.1016/j.jclinepi.2011.01.011

9. Guyatt GH, Oxman AD, Kunz R, et al. GRADE guidelines: 7. Rating the quality of evidence—inconsistency. *J Clin Epidemiol* 2011;64(12):1294-302. doi: 10.1016/j.jclinepi.2011.03.017

10. Guyatt GH, Oxman AD, Kunz R, et al. GRADE guidelines: 8. Rating the quality of evidence—indirectness. *J Clin Epidemiol* 2011;64(12):1303-10. doi: 10.1016/j.jclinepi.2011.04.014

11. Guyatt GH, Oxman AD, Sultan S, et al. GRADE guidelines: 9. Rating up the quality of evidence. *J Clin Epidemiol* 2011;64(12):1311-16. doi: 10.1016/j.jclinepi.2011.06.004

12. Alonso-Coello P, Schünemann HJ, Moberg J, et al. GRADE Evidence to Decision (EtD) frameworks: a systematic and transparent approach to making well informed healthcare choices. 1: Introduction. *BMJ* 2016;353:i2016. doi: 10.1136/bmj.i2016

13. Alonso-Coello P, Oxman AD, Moberg J, et al. GRADE Evidence to Decision (EtD) frameworks: a systematic and transparent approach to making well informed healthcare choices. 2: Clinical practice guidelines. *BMJ* 2016;353:i2089. doi: 10.1136/bmj.i2089

14. Traversy G, Barnieh L, Akl EA, et al. Managing conflicts of interest in the development of health guidelines. *Canadian Medical Association Journal* 2021;193(2):E49-E54. doi: 10.1503/cmaj.200651

15. Vos T, Lim SS, Abbafati C, et al. Global burden of 369 diseases and injuries in 204 countries and territories, 1990–2019: a systematic analysis for the Global Burden of Disease Study 2019. *The Lancet* 2020;396(10258):1204-22. doi: 10.1016/S0140-6736(20)30925-9

16. Ezzati M, Riboli E. Behavioral and Dietary Risk Factors for Noncommunicable Diseases. *N Engl J Med* 2013;369(10):954-64. doi: 10.1056/NEJMra1203528

17. Schienkiewitz A, Mensink GBM, Kuhnert R, et al. Overweight and obesity among adults in Germany. *Journal of Health Monitoring* 2017;2(2):20-27. doi: 10.17886/RKI-GBE-2017-038

18. Schienkiewitz A, Brettschneider A-K, Damerow S, et al. Overweight and obesity among children and adolescents in Germany. Results of the cross-sectional KiGGS Wave 2 study and trends. *Journal of Health Monitoring* 2018;3(1):15-22. doi: 10.17886/RKI-GBE-2018-022

19. Singh GM, Micha R, Khatibzadeh S, et al. Estimated Global, Regional, and National Disease Burdens Related to Sugar-Sweetened Beverage Consumption in 2010. *Circulation* 2015;132(8):639-66. doi: 10.1161/CIRCULATIONAHA.114.010636

20. Schwingshackl L, Knüppel S, Michels N, et al. Intake of 12 food groups and disability-adjusted life years from coronary heart disease, stroke, type 2 diabetes, and colorectal cancer in 16 European countries. *Eur J Epidemiol* 2019;34(8):765-75. doi: 10.1007/s10654-019-00523-4

21. Singh GM, Micha R, Khatibzadeh S, et al. Global, Regional, and National Consumption of Sugar-Sweetened Beverages, Fruit Juices, and Milk: A Systematic Assessment of Beverage Intake in 187 Countries. *PloS One* 2015;10(8):e0124845-e45. doi: 10.1371/journal.pone.0124845

22. Della Corte K, Fife J, Gardner A, et al. World trends in sugar-sweetened beverage and dietary sugar intakes in children and adolescents: a systematic review. *Nutrition Reviews* 2021;79(3):274-88. doi: 10.1093/nutrit/nuaa070

23. Duffey KJ, Huybrechts I, Mouratidou T, et al. Beverage consumption among European adolescents in the HELENA study. *European Journal of Clinical Nutrition* 2012;66(2):244-52. doi: 10.1038/ejcn.2011.166

24. Cabrera Escobar MA, Veerman JL, Tollman SM, et al. Evidence that a tax on sugar sweetened beverages reduces the obesity rate: a meta-analysis. *BMC Public Health* 2013;13(1):1072. doi: 10.1186/1471-2458-13-1072

25. Teng AM, Jones AC, Mizdrak A, et al. Impact of sugar-sweetened beverage taxes on purchases and dietary intake: Systematic review and meta-analysis. *Obes Rev* 2019;20(9):1187-204. doi: 10.1111/obr.12868

26. Itria A, Borges SS, Rinaldi AEM, et al. Taxing sugar-sweetened beverages as a policy to reduce overweight and obesity in countries of different income classifications: a systematic review. *Public Health Nutr* 2021;24(16):5550-60. doi: 10.1017/s1368980021002901

27. von Philipsborn P, Stratil JM, Burns J, et al. Environmental interventions to reduce the consumption of sugar-sweetened beverages and their effects on health. *Cochrane Database Syst Rev* 2019(6) doi: 10.1002/14651858.CD012292.pub2

28. Ernst JB, Arens-Azevêdo U, Bitzer B, et al. Quantitative Empfehlung zur Zuckerzufuhr in Deutschland. Bonn: Deutsche Adipositas-Gesellschaft, Deutsche Diabetes Gesellschaft und Deutsche Gesellschaft für Ernährung 2018. [Available from: https://www.dge.de/fileadmin/public/doc/ws/stellungnahme/Konsensuspapier_Zucker_DAG_DDG_DGE_2018.pdf.

29. Eykelenboom M, Olthof MR, van Stralen MM, et al. The effects of a sugar-sweetened beverage tax and a nutrient profiling tax based on Nutri-Score on consumer food purchases in a virtual supermarket: a randomised controlled trial. *Public Health Nutr* 2021:1-13. doi: 10.1017/S1368980021004547

30. Pell D, Mytton O, Penney TL, et al. Changes in soft drinks purchased by British households associated with the UK soft drinks industry levy: controlled interrupted time series analysis. *BMJ* 2021;372:n254. doi: 10.1136/bmj.n254

31. Gibson LA, Lawman HG, Bleich SN, et al. No Evidence of Food or Alcohol Substitution in Response to a Sweetened Beverage Tax. *Am J Prev Med* 2021;60(2):e49-e57. doi: 10.1016/j.amepre.2020.08.021

32. Kurz CF, König AN. The causal impact of sugar taxes on soft drink sales: evidence from France and Hungary. *Eur J Health Econ* 2021;22(6):905-15. doi: 10.1007/s10198-021-01297-x

33. Toews I, Lohner S, Küllenberg de Gaudry D, et al. Association between intake of non-sugar sweeteners and health outcomes: systematic review and meta-analyses of randomised and non-

randomised controlled trials and observational studies. *BMJ* 2019;364:k4718. doi: 10.1136/bmj.k4718

34. Laviada-Molina H, Molina-Segui F, Pérez-Gaxiola G, et al. Effects of nonnutritive sweeteners on body weight and BMI in diverse clinical contexts: Systematic review and meta-analysis. *Obes Rev* 2020;21(7):e13020. doi: 10.1111/obr.13020

35. Petticrew M, Knai C, Thomas J, et al. Implications of a complexity perspective for systematic reviews and guideline development in health decision making. *BMJ Global Health* 2019;4(Suppl 1):e000899. doi: 10.1136/bmjgh-2018-000899

36. Powell LM, Wada R, Persky JJ, et al. Employment impact of sugar-sweetened beverage taxes. *Am J Public Health* 2014;104(4):672-7. doi: 10.2105/ajph.2013.301630

37. Afshin A, Peñalvo JL, Del Gobbo L, et al. The prospective impact of food pricing on improving dietary consumption: A systematic review and meta-analysis. *PLoS One* 2017;12(3):e0172277. doi: 10.1371/journal.pone.0172277

38. Pfinder M, Heise TL, Hilton Boon M, et al. Taxation of unprocessed sugar or sugar-added foods for reducing their consumption and preventing obesity or other adverse health outcomes. *Cochrane Database Syst Rev* 2020;4(4):Cd012333. doi: 10.1002/14651858.CD012333.pub2

39. Heise TL, Katikireddi SV, Pega F, et al. Taxation of sugar-sweetened beverages for reducing their consumption and preventing obesity or other adverse health outcomes. *Cochrane Database of Systematic Reviews* 2016(8) doi: 10.1002/14651858.CD012319

40. Te Morenga L, Mallard S, Mann J. Dietary sugars and body weight: systematic review and meta-analyses of randomised controlled trials and cohort studies. *BMJ* 2013;346:e7492. doi: 10.1136/bmj.e7492

41. Schwingshackl L, Hoffmann G, Lampousi AM, et al. Food groups and risk of type 2 diabetes mellitus: a systematic review and meta-analysis of prospective studies. *Eur J Epidemiol* 2017;32(5):363-75. doi: 10.1007/s10654-017-0246-y

42. Schwingshackl L, Schwedhelm C, Hoffmann G, et al. Food groups and risk of all-cause mortality: a systematic review and meta-analysis of prospective studies. *Am J Clin Nutr* 2017;105(6):1462-73. doi: 10.3945/ajcn.117.153148

43. Bechthold A, Boeing H, Schwedhelm C, et al. Food groups and risk of coronary heart disease, stroke and heart failure: A systematic review and dose-response meta-analysis of prospective studies. *Crit Rev Food Sci Nutr* 2019;59(7):1071-90. doi: 10.1080/10408398.2017.1392288

44. Schwingshackl L, Schwedhelm C, Hoffmann G, et al. Food groups and risk of colorectal cancer. *Int J Cancer* 2018;142(9):1748-58. doi: 10.1002/ijc.31198

45. Schwingshackl L, Schwedhelm C, Hoffmann G, et al. Food Groups and Risk of Hypertension: A Systematic Review and Dose-Response Meta-Analysis of Prospective Studies. *Adv Nutr* 2017;8(6):793-803. doi: 10.3945/an.117.017178

46. Schlesinger S, Neuenschwander M, Schwedhelm C, et al. Food Groups and Risk of Overweight, Obesity, and Weight Gain: A Systematic Review and Dose-Response Meta-Analysis of Prospective Studies. *Adv Nutr* 2019;10(2):205-18. doi: 10.1093/advances/nmy092

47. Grade Definitions. U.S. Preventive Services task Force. Oktober 2018. [Available from: https://www.uspreventiveservicestaskforce.org/uspstf/about-uspstf/methods-and-processes/grade-definitions.

48. Martin CB, Herrick KA, Sarafrazi N, et al. Attempts to lose weight among adults in the United States, 2013-2016. *NCHS Data Brief* 2018;313

49. AARP and National Alliance for Caregiving. Caregiving in the United States 2020. Washington, DC: AARP, 2020.

50. Withrow D, Alter DA. The economic burden of obesity worldwide: a systematic review of the direct costs of obesity. *Obes Rev* 2011;12(2):131-41. doi: 10.1111/j.1467-789X.2009.00712.x

51. Effertz T, Engel S, Verheyen F, et al. The costs and consequences of obesity in Germany: a new approach from a prevalence and life-cycle perspective. *Eur J Health Econ* 2016;17(9):1141-58. doi: 10.1007/s10198-015-0751-4

52. Saxena A, Stacey N, Puech PDR, et al. The distributional impact of taxing sugar-sweetened beverages: findings from an extended cost-effectiveness analysis in South Africa. *BMJ Glob Health* 2019;4(4):e001317. doi: 10.1136/bmjgh-2018-001317

53. Kao KE, Jones AC, Ohinmaa A, et al. The health and financial impacts of a sugary drink tax across different income groups in Canada. *Econ Hum Biol* 2020;38:100869. doi: 10.1016/j.ehb.2020.100869

54. Wilde P, Huang Y, Sy S, et al. Cost-Effectiveness of a US National Sugar-Sweetened Beverage Tax With a Multistakeholder Approach: Who Pays and Who Benefits. *Am J Public Health* 2018;109(2):276-84. doi: 10.2105/AJPH.2018.304803

55. Lee Y, Mozaffarian D, Sy S, et al. Health Impact and Cost-Effectiveness of Volume, Tiered, and Absolute Sugar Content Sugar-Sweetened Beverage Tax Policies in the United States: A Microsimulation Study. *Circulation* 2020;142(6):523-34. doi: 10.1161/circulationaha.119.042956

56. Bolt-Evensen K, Vik FN, Stea TH, et al. Consumption of sugar-sweetened beverages and artificially sweetened beverages from childhood to adulthood in relation to socioeconomic status – 15 years follow-up in Norway. *Int J Behav Nutr Phys Act* 2018;15(1):8. doi: 10.1186/s12966-018-0646-8

57. Sommer I, Griebler U, Mahlknecht P, et al. Socioeconomic inequalities in non-communicable diseases and their risk factors: an overview of systematic reviews. *BMC Public Health* 2015;15(1):914. doi: 10.1186/s12889-015-2227-y

58. Backholer K, Sarink D, Beauchamp A, et al. The impact of a tax on sugar-sweetened beverages according to socio-economic position: a systematic review of the evidence. *Public Health Nutr* 2016;19(17):3070-84. doi: 10.1017/s136898001600104x

59. Jain V, Crosby L, Baker P, et al. Distributional equity as a consideration in economic and modelling evaluations of health taxes: A systematic review. *Health Policy* 2020;124(9):919-31. doi: 10.1016/j.healthpol.2020.05.022

60. Djojosoeparto SK, Eykelenboom M, Poelman MP, et al. Stakeholder views on the potential impact of a sugar-sweetened beverages tax on the budgets, dietary intake, and health of lower and higher socioeconomic groups in the Netherlands. *Arch Public Health* 2020;78(1):125. doi: 10.1186/s13690-020-00507-x

61. Lal A, Mantilla-Herrera AM, Veerman L, et al. Modelled health benefits of a sugar-sweetened beverage tax across different socioeconomic groups in Australia: A cost-effectiveness and equity analysis. *PLoS Med* 2017;14(6):e1002326. doi: 10.1371/journal.pmed.1002326

62. Krieger J, Magee K, Hennings T, et al. How sugar-sweetened beverage tax revenues are being used in the United States. *Preventive Medicine Reports* 2021;23:101388. doi: 10.1016/j.pmedr.2021.101388

63. Eykelenboom M, van Stralen MM, Olthof MR, et al. Political and public acceptability of a sugar-sweetened beverages tax: a mixed-method systematic review and meta-analysis. *Int J Behav Nutr Phys Act* 2019;16(1):78. doi: 10.1186/s12966-019-0843-0

64. Eykelenboom M, van Stralen MM, Olthof MR, et al. Public acceptability of a sugar-sweetened beverage tax and its associated factors in the Netherlands. *Public Health Nutr* 2021;24(8):2354-64. doi: 10.1017/S1368980020001500

65. Elstein JG, Lowery CM, Sangoi P, et al. Analysis of Public Testimony About Philadelphia's Sweetened Beverage Tax. *Am J Prev Med* 2021 doi: 10.1016/j.amepre.2021.08.023

66. Lancsar E, Ride J, Black N, et al. Social acceptability of standard and behavioral economic inspired policies designed to reduce and prevent obesity. *Health Econ* 2022;31(1):197-214. doi: 10.1002/hec.4451

67. Obesity Evidence Hub. Countries that have taxes on sugar-sweetened beverages (SSBs): Cancer Counciol; 2021 [updated 17.08.2021. Available from: https://www.obesityevidencehub.org.au/collections/prevention/countries-that-have-implemented-taxes-on-sugar-sweetened-beverages-ssbs.

68. World Health Organisation. Fiscal policies for diet and prevention of noncommunicable diseases : technical meeting report, 5-6 May 2015, Geneva, Switzerland; 2016 [Available from: http://apps.who.int/iris/bitstream/10665/250131/1/9789241511247-eng.pdf.

69. Pomeranz JL, Wilde P, Huang Y, et al. Legal and Administrative Feasibility of a Federal Junk Food and Sugar-Sweetened Beverage Tax to Improve Diet. *Am J Public Health* 2018;108(2):203-09. doi: 10.2105/AJPH.2017.304159

70. Eykelenboom M, Djojosoeparto SK, van Stralen MM, et al. Stakeholder views on taxation of sugar-sweetened beverages and its adoption in the Netherlands. *Health Promot Int* 2021 doi: 10.1093/heapro/daab114

71. George A. Not so sweet refrain: sugar-sweetened beverage taxes, industry opposition and harnessing the lessons learned from tobacco control legal challenges. *Health Econ Policy Law* 2019;14(4):509-35. doi: 10.1017/S1744133118000178
